# Cancer Cachexia and Antitumor Immunity: Common Mediators and Potential Targets for New Therapies

**DOI:** 10.3390/life12060880

**Published:** 2022-06-12

**Authors:** Konstantinos Rounis, Dimitrios Makrakis, Ioannis Gioulbasanis, Simon Ekman, Luigi De Petris, Dimitris Mavroudis, Sofia Agelaki

**Affiliations:** 1 Department of Medical Oncology, University General Hospital, 71110 Heraklion, Crete, Greece; dmakrak@uw.edu (D.M.); mavroudis@uoc.gr (D.M.); agelakisofia@gmail.com (S.A.); 2 Theme Cancer, Karolinska University Hospital/Department of Oncology-Pathology, Karolinska Institutet, 17164 Stockholm, Sweden; simon.ekman@ki.se (S.E.); luigi.depetris@ki.se (L.D.P.); 3 Department of Medicine, Division of Oncology, University of Washington, Seattle, WA 98195, USA; 4 Department of Medical Oncology, Animus Kyanus Stavros General Clinic, 41222 Larissa, Thessaly, Greece; rodopatis@gmail.com; 5 Laboratory of Translational Oncology, School of Medicine, University of Crete, 70013 Heraklion, Crete, Greece

**Keywords:** immunotherapy, cancer cachexia, resistance to immunotherapy, PD-1, antitumor immunity, tumor microenvironment, cytokines, cachexia pathogenesis, immune checkpoint inhibitors

## Abstract

Cancer cachexia syndrome (CCS) is a multifactorial metabolic syndrome affecting a significant proportion of patients. CCS is characterized by progressive weight loss, alterations of body composition and a systemic inflammatory status, which exerts a major impact on the host’s innate and adaptive immunity. Over the last few years, the development of immune checkpoint inhibitors (ICIs) transformed the treatment landscape for a wide spectrum of malignancies, creating an unprecedented opportunity for long term remissions in a significant subset of patients. Early clinical data indicate that CCS adversely impairs treatment outcomes of patients receiving ICIs. We herein reviewed existing evidence on the potential links between the mechanisms that promote the catabolic state in CCS and those that impair the antitumor immune response. We show that the biological mediators and processes leading to the development of CCS may also participate in the modulation and the sustainment of an immune suppressive tumor microenvironment and impaired anti-tumor immunity. Moreover, we demonstrate that the deregulation of the host’s metabolic homeostasis in cancer cachexia is associated with resistance to ICIs. Further research on the interrelation between cancer cachexia and anti-tumor immunity is required for the effective management of resistance to immunotherapy in this specific but large subgroup of ICI treated individuals.

## 1. Background

Cachexia has been recognized as a distinct syndrome associated with weight, muscle, and adipose tissue loss and a negative shift in the homeostatic equilibrium between energy and protein balance, which cannot be reversed by the provision of adequate nutrients [1]. The cachexia syndrome may accompany malignant as well as non-malignant diseases, such as heart failure, chronic obstructive pulmonary disorder and AIDS. Cancer cachexia syndrome (CCS) has a high prevalence, occurring in up to 80% of end-stage cancer patients, and it has been associated with increased complications after surgical interventions, reduced responsiveness and increased toxicity to chemotherapy, and overall adverse outcomes [2]. Moreover, it is further related to a progressive deterioration of performance status and a poor quality of life, and it directly accounts for approximately 20% of cancer deaths [1].

CCS is characterized by a complex underlying pathophysiology elicited by numerous soluble mediators, produced either directly by tumor cells or by their interaction with cells of the tumor microenvironment (TME) and the host’s immune system. These mediators are responsible for the development of a systemic inflammatory response, activation of unprofitable biochemical cycles, and disruption of physiological endocrine and metabolic processes [3]. Cachexia is not an inevitable consequence of cancer, and it seems to depend on the activation and the perpetual sustainment of an underlying inflammatory response leading to the disrupted homeostasis of the mechanisms that calibrate metabolic pathways [1,3]. In addition, the underlying systemic inflammatory process observed in CCS has an impact on the innate and the adaptive immune system function at a multilayer level since patients with cancer cachexia suffer from increased susceptibility to infections [4].

Anti-cancer immunotherapy with immune checkpoint inhibitors (ICIs) or with sophisticated cell-based therapies like Chimeric Antigen Receptor (CAR)-T cells have revolutionized daily clinical practice as they offer the opportunity for long term remissions or even cure to a subset of patients. However, only a proportion of patients treated with ICIs, either as a monotherapy or in combinations, will derive significant clinical benefit, and despite intensive research efforts, the mechanisms of primary or secondary resistance to immunotherapy have not as yet been completely elucidated [5]. The understanding of key mechanisms regulating this response heterogeneity represents a highly unmet need and a field of ongoing intensive research.

The underlying inflammatory process and the disruption of metabolic homeostatic circuits that occur in individuals with CCS may result in the perturbation of anti-tumor immune interactions. Furthermore, clinical evidence is being progressively gathered on the adverse role of cancer cachexia and sarcopenia on the outcome of cancer patients treated with ICIs [6,7,8]. Considering the high prevalence of CCS amongst cancer patients and the widespread use of ICIs for cancer therapy, the question of the underlying mechanisms exerting an immune suppressive effect on patients with CCS and the envision of novel approaches to bypass these mechanisms represents a topic of high importance. We herein present a review of the current knowledge to uncover potential links between the mechanisms underlying the catabolic changes in CCS and those of impaired antitumor immunity. As such, we summarized the available preclinical and clinical data highlighting the clinical importance of further investigations on the molecular pathogenesis of CCS, which could potentially help us decipher the mechanisms of immunotherapy resistance. Such research focus could result in a better understanding of the current biomarkers for the prediction of immunotherapy clinical outcomes as well as to the development of novel approaches for the management of resistance to ICIs.

## 2. Common Mediators of Cachexia and the Cancer-Immunity Cycle

**Figure 1:**
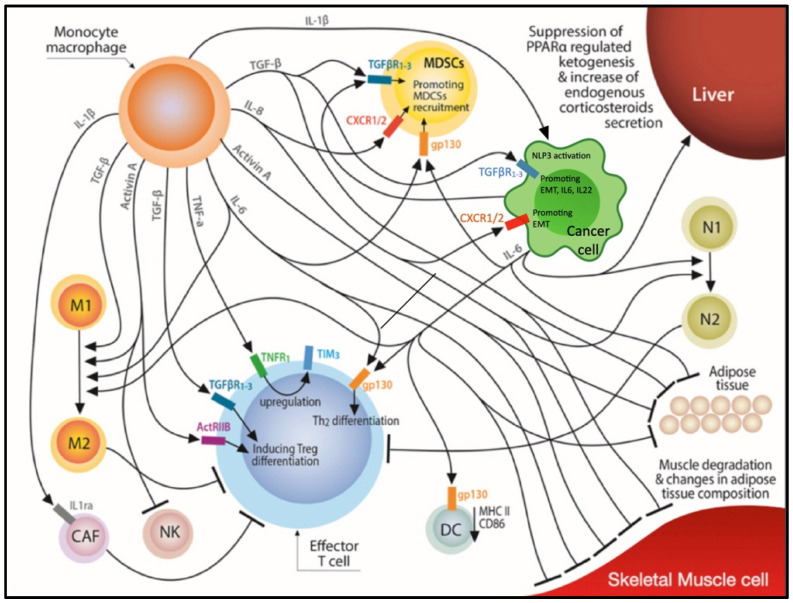
The complexity of effects induced by a plethora of cachexia mediators leading to immune suppression and alteration in muscle and adipose tissue composition. Abbreviations: ActRIIB: Τype IIβ Activin receptor, CAF: Cancer associated fibroblast, CXCR1/2: C-X-C motif chemokine receptor 1–2, DC: Dendritic cell, EMT: Epithelial-to-mesenchymal transition, gp130: Glycoprotein 130, MDSC: Myeloid derived suppressor cell, M1 and M2: M1 and M2 macrophage subtypes, N1 and N2: N1 and N2 tumor infiltrating neutrophils subtypes, NK: Natural killer cell, NLP3: Nodule inception protein-like protein 3, PPARα: Peroxisome proliferator-activated receptor alpha, TGFβR1-3: Transforming growth factor beta receptors 1–3, TIM3: T-cell immunoglobulin and mucin-domain containing-3, TNFR1: Tumor necrosis factor receptor 1.

A long list of serum factors secreted by tumor or immune cells has been shown in experimental models to have the potential to induce a cachexia phenotype [1,3]. Several of those, such as members of the TNF-α superfamily, Interleukins (IL)-1α, -1β, -6, -8 and members of the Transforming Growth Factor (TGF)—β superfamily, have been also recognized to affect anti-cancer immunity. The complex interplay between the various cachexia mediators and their effect on immune suppression and the modulation of TME is depicted in Figure 1. The dual role of some of these particular mediators is presented below in detail.

### 2.1. Tumor Necrosis Factor Alpha (TNF-α)

TNF-α is an acute phase reactant mainly released by activated macrophages as well as by a number of other immune cells that have been associated with the development of cachexia in the setting of cancer and infectious diseases [9]. TNF-α exerts its catabolic functions in a pleiotropic manner. It promotes muscle wasting by inhibiting myocyte differentiation and stimulating muscle protein degradation via activation of the ubiquitin E3 ligase pathway [10]. It can also cause adipose tissue atrophy through the suppression of the synthesis of adipocyte differentiation transcription factors [11] and increased energy wasting via the elevation of cardiolipin content in liver mitochondria [11]. Finally, TNF-α also operates at the central nervous system (CNS) level and more specifically at the hypothalamus where it can trigger sickness behavior [12]. In addition, through its effect on the CNS, it can induce muscle protein degradation and lipolysis through the stimulation of propiomelanocortin (POMC) and agouti-related protein (AGRP) receptors [12].

In mouse models, the administration of TNF-α has been shown to induce cachexia [13], however TNF inhibition alone was not sufficient to reverse the cachexia phenotype, indicating the complex underlying pathophysiology of the syndrome [14]. Clinical studies addressing the association between serum TNF-α levels and CCS in cancer patients have yielded conflicting results [15,16]. Furthermore, administration of anti-TNF-α monoclonal antibodies in patients with pancreatic and lung cancers failed to demonstrate any benefit in the palliation of cancer cachexia [17,18].

In addition to its catabolic functions, TNF-α-induced activation of TNFR1 has been demonstrated to impair the accumulation of intra-tumoral CD8+ T cells and to up-regulate the co-inhibitory immune checkpoint T-cell immunoglobulin and mucin-domain containing-3 (TIM-3) [19]. In the same study, TNF-α blockade overcame resistance to anti-PD-1 therapy in melanoma mice models [19]. Clinical data have shown that the administration of anti TNF-α monoclonal antibodies due to immune related adverse events in immunotherapy treated melanoma patients did not compromise treatment outcomes [20].

It is possible that continuous immune stimulation by TNF-α may lead to T cell exhaustion, upregulation of secondary immune checkpoints, and treatment failure. A phase Ib trial is currently evaluating the combination of ipilimumab and nivolumab along with the anti-TNF-α antibodies certolizumab or infliximab in patients with advanced melanoma (NCT03293784).

### 2.2. TNF-Related Weak Inducer of Apoptosis (TWEAK)

TWEAK, a pro-cachexia cytokine belonging to the TNF superfamily, interacts through the TNF receptor superfamily member 12A (TNFRSF12A; also known as fibroblast growth factor-inducible 14 (Fn14) and TWEAKR). TWEAK has been recently recognized as a multifunctional cytokine that induces skeletal and cardiac muscle atrophy by activating the ubiquitin proteolytic system and the p50 subunit of the NF-κB signaling pathway, respectively [21]. Tumor oriented Fn14 signaling in experimental mouse models resulted in a clinical phenotype similar to CCS in which the administration of anti-TNFRSF12A antibodies inhibited weight loss [22].

In parallel with its role as a cachexia mediator, TWEAK also functions as a negative regulator of the transition from innate to adaptive Th1 immunity. More specifically, TWEAK binding to Fn14 inhibits signal transducer and activator of transcription protein (STAT)-1 activation and suppresses interferon γ (IFN-γ) and IL-12 production [23]. TWEAK^−/−^ mice demonstrated robust natural killer (NK) cells and Th1 T cells antitumor responses, and they were able to reject B16 melanoma model tumors in contrast with their wild-type (wt) counterparts [23]. In preclinical models, administration of monoclonal antibodies targeting the TWEAK/Fn14 interaction led to tumor shrinkage and enhancement of the host antitumor immune response by attracting immune cells, particularly CD45+ memory cells, within the TME through Monocyte Chemoattractant Protein-1 (MCP-1) activation [24]. There have been so far only two published phase I studies of agents targeting the TWEAK/Fn14 axis in patients with advanced solid tumors yielding modest results [25,26]. A phase I study of RO5458640, a TWEAK antagonist, in patients with advanced solid tumors has completed accrual, and results are yet to be presented (NCT01383733).

Nevertheless, a combination of TWEAK/FN14 inhibition along with PD1/Programmed death-ligand 1 (PD-L1) inhibition has not been tested so far, and it can pose an interesting future strategy. Therefore, TWEAK axis targeting could be further explored both as an antitumor as well as an anti-CCS strategy in patients with cancer.

### 2.3. IL-1α and IL-1β

IL-1α and IL-1β, cytokines of the interleukin 1 family, have also been linked to the development of cachexia and muscle mass depletion in the context of malignancy or infectious diseases. They both exert their actions via binding to the type 1 IL-1 receptor (IL-1R). They are produced mainly by activated macrophages, with other sources being neutrophils and endothelial cells.

IL-1α has multiple roles in the development of CCS. IL-1α exerts its actions mainly via the hypothalamus either directly by binding to POMC and AGRP neurons producing proteolytic and/or lipolytic signals, or indirectly by increasing plasma concentration of tryptophan, which then triggers overproduction of serotonin from hypothalamus causing anorexia and early satiety [27]. However, blockade of IL-1R in mouse models was not sufficient to reverse weight loss presumably for the same reasons that anti-TNF-α monoclonal antibodies also failed to reverse the cachexia phenotype [28].

Preclinical data concerning the effect of IL-1α on antitumor immunity have been conflicting so far. In tumor models of fibrosarcoma and lymphoma, IL-1α overexpression was associated with tumor regression, mainly through the accumulation of intratumoral CD8+ cells [29,30]. On the contrary, other experimental data have reported a tumor promoting effect for IL-1α in pancreatic cancer by maintaining a pro-tumor inflammatory microenvironment through stimulation of cancer associated fibroblasts (CAFs) [31]. Recently, breast tumor-derived IL-1α was reported to act on tumor-infiltrating myeloid cells inducing the expression of thymic stromal lymphopoietin (TSLP), a factor that was crucial for tumor survival and metastatic spreading [32]. The anti-IL-1α monoclonal antibody bermekimab has been tested in a phase III clinical trial in patients with metastatic colorectal cancer refractory to standard treatment with modest results [33]. Further research is needed to clarify the underlying impact of IL-1α on tumorigenesis and disease progression in order to serve as a valid drug target either alone or in combination with other treatments.

Serum IL-1β levels have also been correlated with CCS development and sarcopenia in patients with advanced malignancies [34]. In conjunction with its role as a cachexia mediator, IL-1β impairs immune function in multiple ways including the stimulation of myeloid-derived suppressor cells (MDSCs) [35] and the induction of IL-6 and IL-22 expression [36,37]. Tumors of IL-1β deficient mice demonstrated low levels of macrophages and a relatively high percentage of CD11b+ dendritic cells (DCs), which in turn secreted IL-12 and supported antitumor immunity through the activation of intratumoral CD8+ lymphocytes [38].

The CANTOS trial was a phase III trial designed to test the effect of canakinumab, an anti-IL-1β monoclonal antibody, on the reduction of the risk of subsequent myocardial infarction, non-fatal stroke, or cardiovascular death in patients with previous myocardial infarction and no history of malignancy [39]. An additional analysis of the CANTOS trial revealed that canakinumab reduced the risk of cancer mortality in the group that received 300 mg canakinumab (HR = 0.33, 95% CI: 0.18–0.59), and, importantly, it demonstrated an even more pronounced effect in the reduction of mortality related with lung cancer (HR = 0.23, 95% CI: 0.10–0.54) [40]. These results seem intriguing at first glance but they should be interpreted with caution since they were derived from an exploratory analysis, however, they indicate a potential role for IL-1β in tumorigenesis. IL-1β inhibition with canakimumab is currently being investigated as an anti-tumor strategy in combination with chemotherapy (NCT03626545) or pembrolizumab (NCT03631199) in the metastatic setting or as adjuvant treatment (NCT03447769) in patients with non-small cell lung cancer.

### 2.4. IL-6

IL-6 is a pleiotropic cytokine produced from activated macrophages, and it has an important role in the development of CCS. IL-6 plasma levels have been directly associated with the pathogenesis of CCS and increased mortality in cancer patients [41]. *Apc*^Min/+^ mice, mice that bear a germline mutation in the adenomatous polyposis coli gene (*APC)*, have elevated serum levels of IL-6, and they develop muscle tissue loss and the cancer cachexia phenotype, whereas, *Apc*^Min/+/IL-6−/−^ mice do not. Exogenous administration of IL-6 in *Apc*^Min/+/IL-6−/−^ mice led to the development of the cachexia phenotype, a finding that delineates a possible central role of IL-6 in CCS pathogenesis [42]. IL-6 has been shown in experimental models to induce CCS through various mechanisms such as increased autophagy and upregulation of transcriptional factors that promote myofibrillar protein breakdown [1].

Furthermore, in parallel with its catabolic and muscle wasting effects, IL-6 has a pleiotropic role as a suppressor of antitumor immunity. IL-6 suppresses the dendritic cell (DCs) function and antigen presentation through the inhibition of major histocompatibility complex (MHC)-II and CD86/80 expression [43], directly impairs T cell function through the inhibition of IFN-γ/STAT1 Th1 differentiation [44], and polarizes CD4+T cells to an IL-4 producing Th2 phenotype [45]. Moreover, IL-6 suppresses the formation of effective CD4+ memory cells [46], induces the polarization of macrophages to an M2 phenotype [47], and stimulates the intra-tumoral accumulation of MDSCs [48]. In two mouse models of cachexia, tumor derived IL-6 led to the reprogramming of hepatic metabolism via suppression of peroxisome proliferator-activated receptor alpha (PPARα) regulated ketogenesis that subsequently induced increased endogenous glucocorticoid secretion, which in turn impaired antitumor immunity and resistance to immunotherapy [49]. Dual blockade of the PD-1/PD-L1 axis and IL-6 exerted synergistic effects in melanoma and colorectal mouse models [50,51]. In clinical studies of patients with metastatic melanoma treated with the anti-CTLA-4 monoclonal antibody ipilimumab, lower IL-6 serum levels have been correlated with increased survival [52].

Targeting IL-6 seems to be an attractive approach to enhance tumor response to immunotherapy. There are currently phase Ib-II clinical trials evaluating anti-IL-6 blockade combined with ipilimumab and nivolumab in patients with unresectable or metastatic melanoma (NCT03999749) or with anti-Her2 antibodies in *Her2* amplified breast cancer (NCT03135171).

### 2.5. IL-8

IL-8, a member of the cys-X-cys cytokine family, is a chemokine produced mainly by macrophages and monocytes exerting its function via binding to C-X-C motif chemokine receptor (CXCR) ½ [53]. Elevated serum levels of IL-8 in cancer patients have been associated with the development of CCS and poor survival outcomes [54,55].

In conjunction with its capability to induce an inflammatory and catabolic status, IL-8 has been associated with suppressed antitumor immunity in a plethora of experimental models. IL-8 expression by tumor cells ablates the antitumor immune response through recruitment of N2 tumor associated neutrophils (TANs) [56] and MDSCs [48]. Jin et al. [57] demonstrated that CAR T-cells targeting CXCR1/2 induced chemotaxis of effector T cells within the tumor that lead to complete tumor regression and the development of immunological memory in mouse xenografts models of glioblastoma, ovarian cancer, and pancreatic cancer. Increased serum levels of IL-8 have been correlated with secondary resistance to immunotherapy and disease progression in patients with metastatic melanoma and NSCLC receiving immunotherapy [58].

Based on these available experimental data, the IL-8/CXCR1/2 axis consists of a promising target for the development of a robust antitumor immune response and the enhancement of the activity of currently used immunotherapies. Reparixin, a CXCR1/2 inhibitor was tested in a phase I trial in combination with paclitaxel in patients with triple negative breast cancer (NCT01861054) and a phase II trial is currently ongoing (NCT02370238). A phase I trial of BMS-986253, an anti-IL-8 monoclonal antibody, has been completed [59] and four phase II trials are currently investigating its activity in combination with nivolumab in patients with advanced solid tumors in the metastatic or neoadjuvant setting (NCT04050462, NCT03400332, NCT03689699, NCT04123379).

## 3. Transforming Growth Factor Beta (TGF-β) Family

Members of the TGF-β family have been associated with the development of CCS. Beyond their effects on muscle and adipose tissue composition these molecules also have significant roles as immune modulators.

### 3.1. Activin 

Activin A is a protein complex involved in a wide spectrum of physiologic processes ranging from stimulation of follicle-stimulating hormone (FSH) biosynthesis to cell proliferation, apoptosis, and wound healing [60]. In many catabolic disease states, circulating Activin A levels rise as a paracrine/autocrine factor generated by activated macrophages or certain types of cancer cells [60]. The interaction of Activin A with type Iiβ Activin receptor (ActRIIB) results in muscle degradation and atrophy through subsequent activation of ubiquitin ligases and upregulation of autophagosome formation, and it promotes muscle wasting and cachexia in preclinical models [61]. Pharmacological blockade of the ActRIIB pathway reversed cancer cachexia and muscle wasting, and it led to prolonged survival in preclinical models [62]. In clinical studies, elevated serum levels of Activin A have been associated with the development of CCS and reduced survival in pancreatic cancer patients [63].

In parallel with its catabolic effects, Activin A has also been demonstrated to suppress antitumor immunity through differentiation of CD4+ T cells into T regulatory cells (Tregs) in vitro [64] and polarization of TAMs to an M2 phenotype [65]. Interestingly, Activin A/actRIIB interaction blockade using follistatin improved the NK cell function and antitumor immunity, and it slowed melanoma growth in orthotopic mouse models through inhibition of the SMAD2/3 pathway [66]. Results for the activity and safety in human patients of STM 34, an Activin A inhibitor, were recently published [67]. Therefore, the Activin A/ActRIIB signaling pathway seems to be an attractive target both for the treatment of cachexia as well as for the invigoration of an effective antitumor immune response, however further clinical and translational research is needed in the field.

### 3.2. TGF-β

TGF-β has been correlated with the development of cachexia and fibrosis for more than two decades [68]. In preclinical models, TGF-β release into circulation due to osteolysis from bone metastasis in mouse models activates the SMAD3-NADPH oxidase 4 (NOX4)-ryanodine receptor 1 (RyR1) pathway leading to muscle dysfunction and development of cachexia [69]. In addition, elevated serum levels of TGF-β in patients with colorectal cancer were correlated with the development of CCS [70].

TGF-β has a cardinal role as a negative regulator of the antitumor immune response by inducing differentiation of CD4+ T cells to Tregs, acting as a chemoattractant for MDSCs in the TME, inducing macrophage polarization to an M2 phenotype, promoting epithelial to mesenchymal transition (EMT) and excluding effector CD8+ T cells from the tumor parenchyma [71]. TGF-β blockade also showed promising activity in boosting host’s antitumor immunity mainly via suppressing Treg function [72]. Combined blockade of TGF-β and PDL1 exerted a synergistic effect leading to increased numbers of intratumoral effector T cells and robust antitumor response in mouse models [73].

Phase I and II clinical trials are currently under way evaluating the safety and efficacy of anti TGF-β1-2-3 agents either alone or in combination with ICIs (NCT03192345, NCT02699515, NCT02517398, NCT03451773, NCT03315871, NCT03620201, NCT03579472, NCT03524170, NCT04220775, and NCT03436563).

### 3.3. Growth Differentiation Factor 15 (GDF15)

GDF15 is another member of the TGF-β superfamily expressed in low levels in a wide spectrum of tissues under physiologic conditions. The activation of mitogen-activated protein kinase 11 (MAP3K11) by a GDF-15/GDNF family receptor alpha like (GFRAL) interaction has been identified as the key trigger for weight loss in animal models of cancer-related cachexia [74], and increased serum levels of GDF15 have been associated with the development of CCS, anorexia, increased tumor load, and poor survival outcomes in cancer patients [75].

In preclinical models, GDF-15 has been demonstrated as a potent suppressor of antitumor immunity. GDF-15 inhibits dendritic cell maturation in the TME leading to impaired T cell activation [76], whereas, downregulation of GDF-15 using short hairpin RNA (shRNA) in a glioblastoma model resulted in increased T cell infiltration in the TME and increased survival [77]. Similarly, depletion of GDF-15 in orthotopic pancreatic cancer models restored immunosurveillance in the TME resulting in improved tumor control [78]. Blockade of a GDF15/GFRAL/RET interaction with the monoclonal antibody 3P10 resulted in a reversal of cancer cachexia in mouse models [79]. GDF-15 represents a promising target both for the reversal of cancer cachexia and for the enhancement of an antitumor response, however, additional research is needed to further elucidate its potential either as a single therapy or in combination with existing immunotherapies.

## 4. MDSCS, Cancer Associated Fibroblasts and CCS

### 4.1. Myeloid Derived Suppressor Cells (MDSCs)

MDSCs represent a heterogeneous group of cells of myeloid origin linked with the suppression of antitumor immunity [80]. Increased numbers of MDSCs in the serum or in the TME have been associated with the development of CCS in experimental models and cancer patients [81,82]. MDSCs have been proposed to cause CCS via the induction and the sustainment of an underlying inflammatory process that subsequently results in increased energy expenditure and protein turnover [81,82].

MDSCs suppress the antitumor immune response at a multilayer level. They can promote angiogenesis and induce the production of matrix metalloproteinases [80]. In addition, they cause arginine depletion through increased Arg1 (Arginase) activity, and they produce reactive oxygen species (ROS) that subsequently result in T cell anergy and death [80]. Finally, they stimulate intra-tumoral Treg recruitment and macrophage polarization to an M2 phenotype [80]. Interestingly, in a mouse model with conditional targeting of the *Pdcd1* gene, specific PD-1 ablation in myeloid cells had a more pronounced effect on boosting antitumor immunity and restricting tumor growth and progression compared to specific PD1 ablation on T cells [83]. In the same report, a novel role for the PD-1 pathway in determining lineage fate commitment of myeloid cells was demonstrated, as specific PD-1 ablation on myeloid cells or treatment with anti-PD1/PDL1 antibody suppressed further myeloid compartment expansion and sustainment [83]. Our hypothesis is that in a subset of patients the administration of anti PD-1 antibody can potentially lead to the differentiation of the MDSCs and subsequent suppression of the myeloid compartment thus inhibiting the loophole of the sustained underlying inflammation that drives cancer cachexia.

MDSCs targeting is challenging due to the diverse nature of human MDSCs, however it represents one of the most promising approaches to enhance tumor responsiveness to immunotherapy. Whether MDSC depletion can reverse the CCS phenotype or established resistance to immunotherapy is a question that warrants further research.

### 4.2. Cancer Associated Fibroblasts (CAFs)

CAFs are cells of mesodermal origin that constitute part of the TME and they are derived from mesenchymal progenitors or from preexistent fibroblast pools. The transformation of normal fibroblasts into activated CAFs is modulated by a plethora of paracrine and systemic factors such as ROS and IL-6 [84]. The presence of CAFs has not as yet been directly linked with CCS development, however, recent evidence showed that they may be involved in muscle wasting. Fibroblast associated protein α (FAPα) is the marker used for the detection of fibroblasts in tumors and FAPα positive cells have been found in skeletal tissue in mice. Depletion of FAPα cells resulted in a muscle wasting syndrome in experimental mice model mimicking CCS [85]. In addition, CAFs secrete pro-cachexia mediators such as IL-6, TGF-β, and parathyroid hormone related protein (PTHrp). PTHrp is the primary mediator linked with the process of browning of white adipose tissue [86].

CAFs contribute to the generation of an immune suppressing TME through polarization of macrophages to an M2 phenotype; intra-tumoral recruitment of N2 neutrophils; secretion of pro-tumor cytokines TGF-β, IL-6, and C-X-C motif chemokine 12 (CXCL12); and exclusion of T cells from interacting with tumor cells via matrix remodeling [87]. Given their properties to regulate tumor and host metabolism, CAFs might be another aspect of host metabolic dysregulation that occurs with CCS. Further research is needed to better clarify the role of CAFs in CCS development and tumor progression since disruption of CAFs-tumor-immune system crosstalk could potentially be targeted to alter tumor metabolism and to promote the antitumor immune response.

## 5. P-Selectin and Cachexia

Cancer patients who carry the C allele of the rs6136 SNP in the *SELP* gene, encoding for the adhesion molecule P selectin, have a reduced risk of developing CCS [88]; the previously described polymorphism is associated with reduced serum levels of P selectin [89]. Selectins (E-, P-, and L-selectin) are a family of adhesion molecules that exert a central role in the inflammatory response by orchestrating the recruitment of platelets and immune cells in sites of inflammation [90]. P-selectin glycoprotein ligand-1 (PSGL-1) is expressed in all leukocyte populations, and it is the best characterized ligand for all P-,E-, and L-Selectins [90].

Experimental models using (P-, L- and E-) selectin deficient mice have shown the importance of selectins in promoting metastasis and recruiting CD11b+Ly6C+Ly6G+ MDSCs in the TME [91]. In addition, PSGL-1 is an immune checkpoint regulator [92]. In a mouse model of melanoma, PSGL-1 deficient mice (*Selplg*^−/−^) demonstrated an improved antitumor immune response through downregulation of inhibitory checkpoints and increased intra-tumoral accumulation of effector CD44^hi^CD8+ and CD4+ T cells compared to their wt type counterparts [92]. *Selplg*^−/−^ mice also had higher frequencies of IFN-γ and IL-2 producing T cells [92].

**Figure 2:**
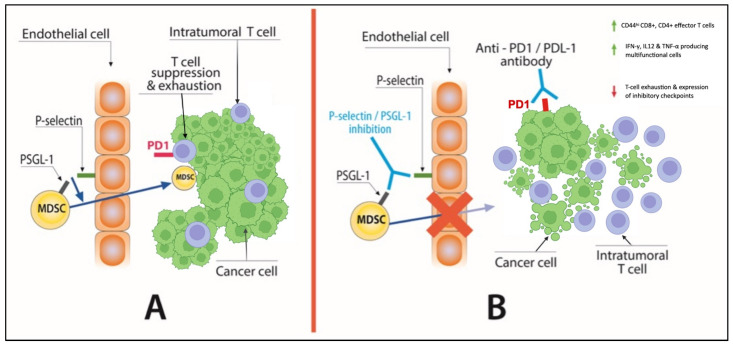
(**A**) P-selectin-PSGL-1 interaction enables the infiltration of the MDSCs in the TME which suppress the antitumor effects of T-cells. (**B**) Inhibition of P-selectin/PSGL-1 interaction can block MDSC cell recruitment in the TME and increase the accumulation of intratumoral effector T cells, thus potentiating the effect of anti-PD-1 treatment. Abbreviations: MDSC: Myeloid derived suppressor cell, PSGL-1: P-selectin glycoprotein ligand 1.

There are no data on the effect of a P Selectin/PSGL1 blockade in the reversal of the CCS phenotype or on the effect of dual inhibition of PD1 or CTLA-4 and the selectin/PSGL1 axis on the antitumor immune response. Further research at a preclinical and a translational level is required for the better understanding of the complex interactions between adhesion molecules, antitumor immunity, and CCS. Nevertheless, the inhibition of the PSGL-1/P selectin pathway seems an attractive target for immunotherapy, especially in patients with preexisting CCS (Figure 2).

## 6. CCS, Autophagy, and Immune Response

Autophagy is a multistep process aiming in the systematic degradation and recycling of cellular components [93]. Autophagy has been linked to the development of the cachexia phenotype and sustained muscle loss in cancer patients in experimental models and translational studies [94,95]. Additionally, tumor derived IL-6 has been implicated as a potential inducer of autophagy in patients with lung and gastrointestinal cancers [96]. Beclin-1 and microtubule-associated proteins 1A/1B light chain 3B II (LC3 B-II), two essential proteins in the formation and function of autophagic machinery, are overexpressed in the skeletal muscle of cancer patients [94]. Autophagy has a dual role in tumorigenesis and progression. Basal autophagy is considered an anti-tumor process, whereas abnormal autophagy promotes the generation and the progression of tumors through abnormal processing and a subsequent accumulation of dysfunctional products of cellular metabolism [97].

Moreover, autophagy represents an essential component of innate and adaptive immunity, especially in the process of antigen presentation and the cross linking of innate and adaptive immune responses, and it exerts a dual role in the shaping of the antitumor response. Autophagosomes engulf intracellular dysregulated proteins and deliver their peptide products to MHC-II containing compartments for the presentation of antigens to a specific CD4+ T cell subset [98]. The autophagic process has the potential to stimulate MHC-I cross-presentation to CD8+ T cells and to attenuate the cross-priming of CD8+ T cells [99]. Finally, autophagy has an important role in the maintenance of the metabolic and oxidative balance of T cells as defective autophagy can lead to impaired degradation of mitochondrial components and a subsequent increased generation of ROS resulting in T cell anergy [100].

On the other hand, autophagy is essential for the survival and immune suppressive function of Tregs, as knockdown of *Atg5* or *Atg7*, two genes essential for the formation and function of autophagy protein complex, leads to Treg death [101]. Furthermore, autophagy deficient Tregs lose expression of the transcription factor forkhead box P3 (FOXP3), especially after activation, and they upregulate metabolic mediators leading to a defective immune suppressive function [101]. Autophagy is essential for the polarization of macrophages to an M2 immune suppressive phenotype [102], and the autophagic process induced by high mobility group box 1 (HMGB1) has been shown to be crucial for the sustainment of MDSCs survival [103].

Despite the fact that autophagy seems to exert an important role in the development of CCS and its dysregulation can be associated with suppression of antitumor immunity, more data are needed to investigate whether the presence of an increased autophagy influx in the skeletal muscle of cancer patients with CCS is associated with an increased autophagy influx in the immune cells of the TME, leading to the accumulation of T-regs and M2 macrophages. If the latter is verified by experimental and translational data, dysregulated autophagy may have a crucial role in primary and/or secondary resistance to checkpoint inhibition and the enzymes of the autophagy machinery may be interesting drug targets for overcoming immunotherapy resistance.

## 7. Cancer Cachexia and Response to Immunotherapy

Published reports have consistently highlighted the fact that lower BMI values, reduced adiposity, and increased catabolism are correlated with reduced efficacy in cancer patients treated with immunotherapy. It should be noted here that these data are mostly retrospective in nature, included patients treated with monotherapies, and used different measures for the classification of the cachexia or sarcopenia status of patients [6,7,104,105,106,107,108].

To investigate the impact of CCS on immunotherapy efficacy, we conducted a prospective trial evaluating the effect of CCS in the therapeutic efficacy of ICIs in patients with metastatic NSCLC [8]. The presence of cancer cachexia (defined as a 5% reduction in body weight within the last 6 months since the initiation of immunotherapy or a ≥ 2% reduction in body weight in patients having a baseline BMI < 20 kg/m^2^ or a reduced muscle mass index according to tomovision analysis) consisted an independent predictor of the increased probability of progression as the best response to immunotherapy [OR = 8.11 (95% CI: 2.95–22.40, *p* < 0.001)] [8]. Furthermore, the presence of baseline cachexia consisted an independent predictor in the multivariate analysis of inferior survival [HR = 2.52 (95% CI: 1.40–2.55, *p* = 0.002)] [8]. A summary of the clinical studies examining the effect of body composition and CCS on the outcome of cancer patients treated with immunotherapy is presented in Table 1.

Despite their limitations, our study along with the other published data converge toward the actuality that CCS can serve as a useful biomarker for the prediction of outcomes of cancer patients treated with immunotherapy. They also highlight that CCS should constitute an additional stratification factor in the design of future clinical trials. A synopsis of the effect on antitumor immunity of the biological parameters that have been identified as pathogenetic factors for cachexia is provided in Table 2. Further research is required to elucidate the association of CCS with immunotherapy efficacy.

## 8. Conclusions

In summary, the data presented above demonstrate that the biological mediators and processes leading to the development of CCS have also been shown to act as suppressors of the antitumor immune response (Table 2). These data suggest that metabolic dysfunction and immune suppression coexist in cancer. Furthermore, the resulting effects from the deregulation of a host’s metabolic homeostasis in cancer cachexia, beyond the previously recognized reduced systemic treatment efficacy and increased toxicity, may include resistance to immune-check point inhibitors. Thus, strategies aiming in the reversal of the catabolic process with anti-cachexia therapies may be necessary to enhance the therapeutic benefit of immunotherapy in patients with CCS.

We suggest that research on CCS pathophysiology and pathogenesis could acquire a novel twist into the investigation of the TME in patients with cancer cachexia in order to identify the specific processes that govern immune suppression in order to decipher the mechanisms that lead to immunotherapy failure in these individuals. Novel immunotherapy approaches could be envisioned for the simultaneous targeting of antitumor immune response and CCS.

Sensitive biomarkers for the identification of earlier and potentially more amenable to therapeutic interventions stages of cachexia are urgently needed. These biomarkers should also be highly recommended as stratification factors in future immunotherapy trials. Finally, translational studies to address patient metabolic status, cachexia, and anti-tumor immunity along the disease trajectory are mandatory to maximize our understanding and to improve patient survival outcomes and quality of life.

## Figures and Tables

**Table 1:** Summary of the clinical studies that examine the effect of cachexia and body composition and treatment outcomes in cancer patients treated with immunotherapy.

Clinical Study	Number (*n*) of Patients	Malignancy Setting	Treatment	Primary Study Point	Results
Turner et al. * [6]	*n* = 1453	Metastatic melanoma and NSCLC	Pembrolizumab	Relationship between Pembrolizumab pharmacokinetics and overall survival	Higher Pembrolizumab clearance (CL_0_) was an adverse prognostic factor for OS and it paralleled disease parameters associated with CCS (multivariate-adjusted CL_0_ HR = **1.64**; 95% CI, 1.06–2.52 for melanoma and HR = **1.88**; 95% CI, 1.22–2.89 for NSCLC).
Naik et al. * [7]	*n* = 139	Metastatic melanoma	Pembrolizumab or Nivolumab or Nivolumab plus Ipilimumab	Association of baseline BMI (at the beginning of immunotherapy) with treatment outcomes	BMI values > 25 kg/m^2^ and <35 kg/m^2^ were a favorable prognostic factor for OS (adjusted-HR: **0.26**; 95% CI:0.1–0.71; *p*-value = **0.008**) and PFS (adjusted-HR: **0.43**; 95% CI: 0.19–0.95; *p*-value: **0.038**) compared to BMI values 18.5–< 25 kg/m^2^
Kichenadasse et al. * [104]	*n* = 2110	Metastatic NSCLC	Atezolizumab	Association of baseline BMI (at the beginning of immunotherapy) with treatment outcomes and adverse events	A linear association between increasing values of BMI and overall survival was observed.
Martini et al. * [105]	*n* = 90	Cancer patients that were treated with immunotherapy in the context of phase I clinical trials in a single center	Immunotherapy based treatments	Association of BMI, subcutaneous fat index (SFI), intermuscular fat index (IFI), and visceral fat index (VFI) with survival outcome.	Patients with an SFI ≥ 73 had a significantly longer OS (hazard ratio, **0.20**; 95% CI, 0.09–0.46 [*p* < **0.001**]) and PFS (hazard ratio, **0.38**; 95% CI, 0.20–0.72 [*p* = **0.003**]) compared with patients with an SFI < 73 and IFI < 3.4 and those with an SFI < 73 and IFI ≥ 3.4)
Shiroyama et al. * [106]	*n* = 42	Previously treated metastatic NSCLC patients	Nivolumab, Pembrolizumab	Association of sarcopenia (calculated by measuring the cross-sectional area of the psoas muscle at the caudal end of the 3rd lumbar verterbrae) with treatment outcomes	Sarcopenia negatively affected PFS (median, 2.1 vs. 6.8 months, *p* = **0.004**) and response rates (40.0% vs. 9.1%, *p* = **0.025**)
Roch et al. * [107]	*n* = 142	Metastatic NSCLC	PD1/PDL1 inhibitors	Effect of cachexia (defined as 5% loss of body within the last 6 months) or the effect of evolving sarcopenia (defined as 5% reduction in skeletal muscle index during treatment) on patient outcomes	Cachexia negatively affected disease control rates (59.9 % vs. 41.1 %, odds ratio: **2.60** (95% CI: 1.03–6.58) and OS HR: **6.26** (95% CI: 2.23–17.57). Evolving sarcopenia was an adverse predictor for shorter PFS, HR: **2.45** (95% CI: 1.09–5.53) and OS, HR: **3.87** (95% CI: 1.60–9.34)
Rounis et al. # [8]	*n* = 83	Metastatic NSCLC	PD1/PDL1	Association of cachexia (defined as weight loss 5% during the last 6 months since the initiation of immunotherapy or any degree of weight loss ≥ 2% and a BMI < 20 kg/m^2^ or reduced muscle mass according to tomovision analysis) with treatment outcomes	The presence of cancer cachexia consisted an independent predictor of increased probability of progression as best response to immunotherapy [OR = **8.11** (95% CI: 2.95–22.40, *p* < **0.001**)] and an independent predictor of inferior survival [HR = **2.52** (95% CI: 1.40–2.55, *p* = **0.002**)]

*: retrospective studies; #: prospective studies.

**Table 2:** Synopsis of the effect of the biological parameters that have been identified as pathogenetic factors for cancer cachexia syndrome on antitumor immunity and of the active clinical trials that evaluate the outcome of their inhibition in conjunction with immunotherapy in cancer patients.

Biological Parameter	Implication on Cachexia Pathogenesis	Adverse Effects on Antitumor Immunity	Positive Effects on Antitumor Immunity	Ongoing Clinical Trials Evaluating the Effect of Inhibition of the Referred Biological Parameter in Combination with Immunotherapy in Cancer Patients
TNF-α	Inhibition of myocyte differentiation and stimulation of protein degradation [10] Inducing adipose tissue atrophy [11] Triggering sickness behavior at hypothalamus [12]	Impairment of intratumoral CD8+ T cells accumulation and upregulation of TIM3 [19]	-	Certolizumab or infliximab in combination with ipilimumab and nivolumab for advanced melanoma (**NCT03293784**)
TWEAK	Inducement of muscle atrophy via activation of ubiquitin proteolytic system [21] TWEAK/Fn14 inhibition reversed cachexia in mouse models [22]	Inhibition of STAT-1 and suppression of IFN-γ and IL-12 [23] Tweak^−/−^ mice exhibit increased numbers of NK and Th1 cells [23] Tweak inhibition lead to tumor shrinkage and accumulation of CD45+ cells in the TME [24]	-	-
IL-1α	Hypothalamus stimulation that leads to proteolytic, lipolytic signals and causes anorexia and early satiety through increased tryptophan plasma levels [27]	Maintenance of tumor suppressive TME through interactions with CAFs [31] Inducing TLSP expression on tumor-infiltrating myeloid cells [32]	IL1α administration resulted in regression in mouse models of lymphoma and fibrosarcoma via accumulation of intratumoral CD8+ T cells [29,30]	-
IL-1β	Increased levels of IL-1β have been associated with cachexia in patients with advanced malignancies [34]	Stimulation of MDSCs [35] Induction of IL-6 and IL-22 expression [36,37] IL1-β deficient mice exhibited improved antitumor immunity compared to wt ones [38]		Canakimumab in combination with pembrolizumab for NSCLC in the metastatic (**NCT03631199**) or the adjuvant setting (**NCT03447769**)
IL-6	Liver stimulation for inducing an acute phase response [1] Upregulation of transcriptional factors that promote myofibrilar breakdown [1] Exogenous administration of IL-6 in *Apc*^Min/+/IL-6−/−^ mice resulted to the development of a cachexia phenotype [42] Induction of autophagy in skeletal muscle [1] Clinical studies have correlated circulating IL-6 levels with the development of CCS in cancer patients [41]	Reprogramming of hepatic metabolism via suppression of peroxisome proliferator-activated receptor alpha (PPARα) regulated ketogenesis that subsequently induced increased endogenous glucocorticoid secretion leading to impaired antitumor immunity and resistance to immunotherapy in two mouse models of cachexia [49] Suppressing DC function through inhibition of MHC-II and CD80/86 [43] Suppressing T cell function through inhibition of IFN-γ/STAT1 Th1 differentiation [44,45] Suppressing the formation of CD4+ memory cells [46] Macrophage polarization to an M2 phenotype [47] Stimulation of MDSCs [48]	-	Tocilizumab in combination with ipilimumab and nivolumab in patients with unresectable or metastatic melanoma (**NCT03999749**) Tocilizumab in combination with trastuzumab and pertuzumab in patients with *Her2* amplified metastatic breast cancer resistant to trastuzumab (**NCT03135171**)
IL-8	Elevated circulating levels of IL-8 have been correlated with the development of CCS in cancer patients [54,55]	Recruitment of N2 TANs [56] Recruitment of MDSCs [48] Inhibition of IL-8/CXCR1/2 pathway in experimental models exerts antitumor effects [57] Increased serum levels of IL-8 have been correlated with secondary resistance to immunotherapy and disease progression in patients with metastatic melanoma and NSCLC receiving immunotherapy [58]	-	BMS-986253 in combination with nivolumab for hormone sensitive prostate cancer (**NCT03689699**), in combination with nivolumab or cabiralizumab in patients with HCC (**NCT04050462**) and in combination with nivolumab in patients with advanced cancer (**NCT03400332**) Neoadjuvant Nivolumab combined with CCR2/5-inhibitor or BMS-986253 for NSCLC or HCC (**NCT04123379**)
Activin A	Activin A causes muscle degradation and atrophy through downstream activation of Atrogin 1 and UBR2 and autophagosome formation [61] Pharmacological blockade of Activin A/ActRIIB pathway reversed cancer cachexia and muscle wasting in preclinical models [62] Elevated serum levels of Activin A have been associated with the development of CCS in pancreatic cancer patients [63]	Activin A has been shown to be able to differentiate CD4+ T cells into Tregs in vitro [64] and has the potential to induce polarization of TAMs to an M2 phenotype [65] Activin A/ActRIIB interaction impairs NK cell function via SMAD2/3 signaling and its blockade improved NK cell function and antitumor immunity and slowed melanoma growth in mouse models [66].	-	-
TGF-β	TGF-β release into circulation activates the SMAD3-NOX4-RyR1 pathway leading to muscle dysfunction and development of cachexia in mouse models [69] Elevated serum levels of TGF-β in patients with colorectal cancer were correlated with the development of CCS [70]	TGF-β induces differentiation of CD4+ T cells to Tregs, acts as a chemoattractant for MDSCs in the TME, induces macrophage polarization to an M2 phenotype and promotes EMT [71] TGF-β blockade has shown activity in boosting host’s antitumor immunity mainly via suppressing Treg function [72] Combined blockade of TGF-β and PDL1 had a synergistic effect and lead to a robust antitumor response in mouse models bearing EMT6 tumors [73]	-	SAR439459 in combination with cemiplimab in advanced solid tumors (**NCT03192345**) MSB0011359C in advanced solid tumors (**NCT02699515**, **NCT02517398**), in combination with gemcitabine for pancreatic adenocarcinoma (**NCT03451773**), in combination with PROSTVAC and CV301 in prostate cancer (**NCT03315871**), for stage II/III *Her2* amplified breast cancer (**NCT03620201**), in combination with eribulin for metastatic TNBC (**NCT03579472**), in combination with RT for ER+PR+Her2-breast cancer (**NCT03524170**) and SBRT for locally recurrent head and neck cancer (**NCT04220775**) and as monotherapy for MSI-high advanced solid tumors (**NCT03436563**).
GDF15	GDF-15/GFRAL interaction has been identified as the key trigger for weight loss in animal models of cancer-related cachexia [74] Increased serum levels of GDF15 have been associated with the development of CCS in cancer patients [75]	GDF-15 inhibits dendritic cell maturation in the TME leading to impaired T cell activation [76], Downregulation of GDF-15 using shRNA in a glioblastoma model resulted in increased T cell infiltration in the TME and increased survival [77] Depletion of GDF-15 in orthotopic pancreatic cancer models restored immunosurveillance in the TME resulting in improved tumor control [78].	-	-
MDSCs	Increased numbers of MDSCs in the serum or in the TME have been linked with the development of CCS in multiple experimental models and cancer patients [81,82]	MDSCs suppress antitumor immunity through angiogenesis promotion, production of matrix metalloproteinases, arginine depletion via increased Arg1 activity, ROS production leading to T cell anergy and death, Treg recruitment and expansion and macrophage polarization to an M2 phenotype [80] Specific PD1 ablation in myeloid cells in preclinical tumor models had a more pronounced effect on boosting antitumor immunity compared to specific PD1 ablation on T cells [83]	-	Cabiralizumab in combination with nivolumab for pretreated metastatic pancreatic cancer (NCT03336216)
p-Selectin	A loss-of-function mutation of the gene that encodes for the adhesion molecule P-Selectin (SELP) has been linked with reduced likelihood of developing CCS in the setting of malignancy [88,89]	P-, L- and E-selectin deficient mice have shown the importance of selectins in promoting metastasis and recruiting CD11b+Ly6C+Ly6G+ MDSCs in the TME [91] PSGL-1 ligation on exhausted T cells PSGL-1 due to TCR engagement extinguished ERK and AKT signaling and upregulated PD1 leading to their diminished survival and function [92] *Selplg*^−/−^ mice demonstrated an improved antitumor immune response and increased intratumoral accumulation of effector CD44^hi^CD8+ and CD4+ T cells compared had higher frequencies of IFN-γ and IL-2 producing T cells [92].	-	-

**Infliximab:** anti-TNF-α monoclonal antibody, **Certolizumab:** anti-TNF-α monoclonal antibody, **Ipilimumab:** anti CTLA-4 monoclonal antibody, **Nivolumab:** anti PD1 monoclonal antibody, **Canakimumab:** anti-IL-1β monoclonal antibody, **Tocilizumab:** anti-IL-6 monoclonal antibody, **BMS-986253 (HuMax-IL8):** anti-IL-8 monoclonal antibody, **SAR439459:** anti-TGF-β monoclonal antibody, **Cemiplimab:** anti-PD1 monoclonal antibody, **MSB0011359C:** bifunctional fusion protein comprised of a fully human IgG1 monoclonal antibody against PDL1 fused to the soluble extracellular domain of TGFR-β2, **Cabiralizumab:** anti-CSFR-1 monoclonal antibody.

## References

[1] Baracos V.E., Martin L., Korc M., Guttridge D.C., Fearon K.C.H. (2018). Cancer-associated cachexia. Nat. Rev. Dis. Primers..

[2] Shachar S.S., Williams G.R., Muss H.B., Nishijima T.F. (2016). Prognostic value of sarcopenia in adults with solid tumours: A meta-analysis and systematic review. Eur. J. Cancer.

[3] Fearon K.C.H., Glass D.J., Guttridge D.C. (2012). Cancer cachexia: Mediators, signaling, and metabolic pathways. Cell Metab..

[4] De Matos-Neto E.M., Lima J.D.C.C., de Pereira W.O., Figuerêdo R.G., Riccardi D.M.D.R., Radloff K., Rodrigo X., das Neves R., Camargo G., Linda F. (2015). Systemic Inflammation in Cachexia—Is Tumor Cytokine Expression Profile the Culprit?. Front. Immunol..

[5] Sharma P., Hu-Lieskovan S., Wargo J.A., Ribas A. (2017). Primary, Adaptive, and Acquired Resistance to Cancer Immunotherapy. Cell.

[6] Turner D.C., Kondic A.G., Anderson K.M., Robinson A.G., Garon E.B., Riess J.W., Jain L., Mayawala K., Kang J., Ebbinghaus S.W. (2018). Pembrolizumab Exposure-Response Assessments Challenged by Association of Cancer Cachexia and Catabolic Clearance. Clin. Cancer Res..

[7] Naik G.S., Waikar S.S., Johnson A.E.W., Buchbinder E.I., Haq R., Hodi F.S., Schoenfeld J.D., Ott P.A. (2019). Complex inter-relationship of body mass index, gender and serum creatinine on survival: Exploring the obesity paradox in melanoma patients treated with checkpoint inhibition. J. Immunother. Cancer.

[8] Rounis K., Makrakis D., Tsigkas A.P., Georgiou A., Galanakis N., Papadaki C., Monastirioti A., Vamvakas L., Kalbakis K., Vardakis N. (2021). Cancer cachexia syndrome and clinical outcome in patients with metastatic non-small cell lung cancer treated with PD-1/PD-L1 inhibitors: Results from a prospective, observational study. Transl. Lung Cancer Res..

[9] Tracey K.J., Lowry S.F., Cerami A. (1988). Cachectin: A hormone that triggers acute shock and chronic cachexia. J. Infect. Dis..

[10] Siddiqui J.A., Pothuraju R., Jain M., Batra S.K., Nasser M.W. (2020). Advances in cancer cachexia: Intersection between affected organs, mediators, and pharmacological interventions. Biochim. Biophys. Acta Rev. Cancer.

[11] Peyta L., Jarnouen K., Pinault M., Coulouarn C., Guimaraes C., Goupille C., de Barros J.P.P., Chevalier S., Dumas J.F., Maillot F. (2015). Regulation of hepatic cardiolipin metabolism by TNFα: Implication in cancer cachexia. Biochim. Biophys. Acta.

[12] Braun T., Zhu X., Szumowski M., Scott G.D., Grossberg A., Levasseur P.R., Graham K., Khan S., Damaraju S., Colmers W.F. (2011). Central nervous system inflammation induces muscle atrophy via activation of the hypothalamic-pituitary-adrenal axis. J. Exp. Med..

[13] Sherry B.A., Gelin J., Fong Y., Marano M., Wei H., Cerami A., Lowry S.F., Lundholm K.G., Moldawer L.L. (1989). Anticachectin/tumor necrosis factor-alpha antibodies attenuate development of cachexia in tumor models. FASEB J..

[14] Tisdale M.J. (1997). Biology of cachexia. J. Natl. Cancer Inst..

[15] Karayiannakis A.J., Syrigos K.N., Polychronidis A., Pitiakoudis M., Bounovas A., Simopoulos K. (2001). Serum levels of tumor necrosis factor-alpha and nutritional status in pancreatic cancer patients. Anticancer. Res..

[16] Maltoni M., Fabbri L., Nanni O., Scarpi E., Pezzi L., Flamini E., Riccobon A., Derni S., Pallotti G., Amadori D. (1997). Serum levels of tumour necrosis factor alpha and other cytokines do not correlate with weight loss and anorexia in cancer patients. Support Care Cancer.

[17] Jatoi A., Dakhil S.R., Nguyen P.L., Sloan J.A., Kugler J.W., Rowland K.M., Soori G.S., Wender D.B., Fitch T.R., Novotny P.J. (2007). A placebo-controlled double blind trial of etanercept for the cancer anorexia/weight loss syndrome: Results from N00C1 from the North Central Cancer Treatment Group. Cancer.

[18] Wiedenmann B., Malfertheiner P., Friess H., Ritch P., Arseneau J., Mantovani G., Caprioni F., Van Cutsem E., Richel D., Dewitte M. (2008). A multicenter, phase II study of infliximab plus gemcitabine in pancreatic cancer cachexia. J. Support Oncol..

[19] Bertrand F., Montfort A., Marcheteau E., Imbert C., Gilhodes J., Filleron T., Rochaix P., Andrieu-Abadie N., Levade T., Meyer N. (2017). TNFα blockade overcomes resistance to anti-PD-1 in experimental melanoma. Nat. Commun..

[20] Weber J.S., Antonia S.J., Topalian S.L., Schadendorf D., Larkin J.M., Sznol M., Liu H.Y., Waxman I., Robert C. (2017). Safety Profile of Nivolumab Monotherapy: A Pooled Analysis of Patients with Advanced Melanoma. J. Clin. Oncol..

[21] Padrão A.I., Moreira-Gonçalves D., Oliveira P.A., Teixeira C., Faustino-Rocha A.I., Helguero L., Vitorino R., Santos L.L., Amado F., Duarte J.A. (2015). Endurance training prevents TWEAK but not myostatin-mediated cardiac remodelling in cancer cachexia. Arch. Biochem. Biophys..

[22] Johnston A.J., Murphy K.T., Jenkinson L., Laine D., Emmrich K., Faou P., Weston R., Jayatilleke K.M., Schloegel J., Talbo G. (2015). Targeting of Fn14 Prevents Cancer-Induced Cachexia and Prolongs Survival. Cell.

[23] Maecker H., Varfolomeev E., Kischkel F., Lawrence D., LeBlanc H., Lee W., Hurst S., Danilenko D., Li J., Filvaroff E. (2005). TWEAK attenuates the transition from innate to adaptive immunity. Cell.

[24] Ye S., Fox M.I., Belmar N.A., Sho M., Chao D.T., Choi N., Fang Y., Zhao V., Keller S.F., Starling G.C. (2017). Enavatuzumab, a Humanized Anti-TWEAK Receptor Monoclonal Antibody, Exerts Antitumor Activity through Attracting and Activating Innate Immune Effector Cells. J. Immunol. Res..

[25] Lassen U.N., Meulendijks D., Siu L.L., Karanikas V., Mau-Sorensen M., Schellens J.H., Jonker D.J., Hansen A.R., Simcox M.E., Schostack K.J. (2015). A phase I monotherapy study of RG7212, a first-in-class monoclonal antibody targeting TWEAK signaling in patients with advanced cancers. Clin. Cancer Res..

[26] Lam E.T., Eckhardt S.G., Messersmith W., Jimeno A., O’Bryant C.L., Ramanathan R.K., Weiss G.J., Chadha M., Oey A., Ding H.T. (2018). Phase I Study of Enavatuzumab, a First-in-Class Humanized Monoclonal Antibody Targeting the TWEAK Receptor, in Patients with Advanced Solid Tumors. Mol. Cancer Ther..

[27] McDonald J.J., McMillan D.C., Laird B.J.A. (2018). Targeting IL-1α in cancer cachexia: A narrative review. Curr. Opin. Support Palliat. Care.

[28] Costelli P., Llovera M., Carbó N., García-Martínez C., López-Sorianoq F.J., Argilés J.M. (1995). Interleukin-1 receptor antagonist (IL-1ra) is unable to reverse cachexia in rats bearing an ascites hepatoma (Yoshida AH-130). Cancer Lett..

[29] Douvdevani A., Huleihel M., Zöller M., Segal S., Apte R.N. (1992). Reduced tumorigenicity of fibrosarcomas which constitutively generate IL-1 alpha either spontaneously or following IL-1 alpha gene transfer. Int. J. Cancer.

[30] Voronov E., Weinstein Y., Benharroch D., Cagnano E., Ofir R., Dobkin M., White R.M., Zoller M., Barak V., Segal S. (1999). Antitumor and immunotherapeutic effects of activated invasive T lymphoma cells that display short-term interleukin 1alpha expression. Cancer Res..

[31] Tjomsland V., Spångeus A., Välilä J., Sandström P., Borch K., Druid H., Falkmer S., Falkmer U., Messmer D., Larsson M. (2011). Interleukin 1α sustains the expression of inflammatory factors in human pancreatic cancer microenvironment by targeting cancer-associated fibroblasts. Neoplasia.

[32] Kuan E.L., Ziegler S.F. (2018). A tumor-myeloid cell axis, mediated via the cytokines IL-1α and TSLP, promotes the progression of breast cancer. Nat. Immunol..

[33] Hickish T., Andre T., Wyrwicz L., Saunders M., Sarosiek T., Kocsis J., Nemecek R., Rogowski W., Lesniewski-Kmak K., Petruzelka L. (2017). MABp1 as a novel antibody treatment for advanced colorectal cancer: A randomised, double-blind, placebo-controlled, phase 3 study. Lancet Oncol..

[34] Scheede-Bergdahl C., Watt H.L., Trutschnigg B., Kilgour R.D., Haggarty A., Lucar E., Vigano A. (2012). Is IL-6 the best pro-inflammatory biomarker of clinical outcomes of cancer cachexia?. Clin. Nutr..

[35] Elkabets M., Ribeiro V.S.G., Dinarello C.A., Ostrand-Rosenberg S., Di Santo J., Apte R.N., Vosshenrich C.A.J. (2010). IL-1β regulates a novel myeloid-derived suppressor cell subset that impairs NK cell development and function. Eur. J. Immunol..

[36] Oh K., Lee O.-Y., Park Y., Seo M.W., Lee D.-S. (2016). IL-1β induces IL-6 production and increases invasiveness and estrogen-independent growth in a TG2-dependent manner in human breast cancer cells. BMC Cancer.

[37] Voigt C., May P., Gottschlich A., Markota A., Wenk D., Gerlach I., Voigt S., Stathopoulos G.T., Arendt K.A.M., Heise C. (2017). Cancer cells induce interleukin-22 production from memory CD4+ T cells via interleukin-1 to promote tumor growth. Proc. Natl. Acad. Sci. USA.

[38] Kaplanov I., Carmi Y., Kornetsky R., Shemesh A., Shurin G.V., Shurin M.R., Dinarello C.A., Voronov E., Apte R.N. (2019). Blocking IL-1β reverses the immunosuppression in mouse breast cancer and synergizes with anti-PD-1 for tumor abrogation. Proc. Natl. Acad. Sci. USA.

[39] Ridker P.M., Everett B.M., Thuren T., MacFadyen J.G., Chang W.H., Ballantyne C., Fonseca F., Nicolau J., Koenig W., Anker S.D. (2017). Antiinflammatory Therapy with Canakinumab for Atherosclerotic Disease. N. Engl. J. Med..

[40] Ridker P.M., MacFadyen J.G., Thuren T., Everett B.M., Libby P., Glynn R.J., Ridker P., Lorenzatti A., Krum H., Varigos J. (2017). Effect of interleukin-1β inhibition with canakinumab on incident lung cancer in patients with atherosclerosis: Exploratory results from a randomised, double-blind, placebo-controlled trial. Lancet.

[41] Moses A.G.W., Maingay J., Sangster K., Fearon K.C.H., Ross J.A. (2009). Pro-inflammatory cytokine release by peripheral blood mononuclear cells from patients with advanced pancreatic cancer: Relationship to acute phase response and survival. Oncol. Rep..

[42] Baltgalvis K.A., Berger F.G., Pena M.M.O., Davis J.M., Muga S.J., Carson J.A. (2008). Interleukin-6 and cachexia in ApcMin/+ mice. Am. J. Physiol. Regul. Integr. Comp. Physiol..

[43] Kitamura H., Kamon H., Sawa S.I., Park S.J., Katunuma N., Ishihara K., Murakami M., Hirano T. (2005). IL-6-STAT3 controls intracellular MHC class II alphabeta dimer level through cathepsin S activity in dendritic cells. Immunity.

[44] Diehl S., Anguita J., Hoffmeyer A., Zapton T., Ihle J.N., Fikrig E., Rincón M. (2000). Inhibition of Th1 differentiation by IL-6 is mediated by SOCS1. Immunity.

[45] Tsukamoto H., Fujieda K., Hirayama M., Ikeda T., Yuno A., Matsumura K., Fukuma D., Araki K., Mizuta H., Nakayama H. (2017). Soluble IL6R Expressed by Myeloid Cells Reduces Tumor-Specific Th1 Differentiation and Drives Tumor Progression. Cancer Res..

[46] Haynes L., Eaton S.M., Burns E.M., Randall T.D., Swain S.L. (2003). CD4 T cell memory derived from young naive cells functions well into old age, but memory generated from aged naive cells functions poorly. Proc. Natl. Acad. Sci. USA.

[47] Zhou J., Qu Z., Sun F., Han L., Li L., Yan S., Stabile L.P., Chen L.-F., Siegfried J.M., Xiao G. (2017). Myeloid STAT3 Promotes Lung Tumorigenesis by Transforming Tumor Immunosurveillance into Tumor-Promoting Inflammation. Cancer Immunol. Res..

[48] Gabrilovich D.I., Nagaraj S. (2009). Myeloid-derived suppressor cells as regulators of the immune system. Nat. Rev. Immunol..

[49] Flint T.R., Janowitz T., Connell C.M., Roberts E., Denton A., Coll A.P., Jodrell D.I., Fearon D.T. (2016). Tumor-Induced IL-6 Reprograms Host Metabolism to Suppress Anti-tumor Immunity. Cell Metab..

[50] Tsukamoto H., Fujieda K., Miyashita A., Fukushima S., Ikeda T., Kubo Y., Senju S., Ihn H., Nishimura Y., Oshiumi H. (2018). Combined Blockade of IL6 and PD-1/PD-L1 Signaling Abrogates Mutual Regulation of Their Immunosuppressive Effects in the Tumor Microenvironment. Cancer Res..

[51] Li J., Xu J., Yan X., Jin K., Li W., Zhang R. (2018). Targeting Interleukin-6 (IL-6) Sensitizes Anti-PD-L1 Treatment in a Colorectal Cancer Preclinical Model. Med. Sci. Monit..

[52] Damuzzo V., Solito S., Pinton L., Carrozzo E., Valpione S., Pigozzo J., Giancristofaro R.A., Chiarion-Sileni V., Mandruzzato S. (2016). Clinical implication of tumor-associated and immunological parameters in melanoma patients treated with ipilimumab. Oncoimmunology.

[53] Dixit N., Simon S.I. (2012). Chemokines, selectins and intracellular calcium flux: Temporal and spatial cues for leukocyte arrest. Front. Immunol..

[54] Gioulbasanis I., Patrikidou A., Kitikidou K., Papadimitriou K., Vlachostergios P.J., Tsatsanis C., Margioris A.N., Papandreou C.N., Mavroudis D., Georgoulias V. (2012). Baseline plasma levels of interleukin-8 in stage IV non-small-cell lung cancer patients: Relationship with nutritional status and prognosis. Nutr. Cancer..

[55] Hou Y.-C., Wang C.-J., Chao Y.-J., Chen H.-Y., Wang H.-C., Tung H.-L., Lin J.-T., Shan Y.-S. (2018). Elevated Serum Interleukin-8 Level Correlates with Cancer-Related Cachexia and Sarcopenia: An Indicator for Pancreatic Cancer Outcomes. J. Clin. Med..

[56] Mishalian I., Bayuh R., Eruslanov E., Michaeli J., Levy L., Zolotarov L., Singhal S., Albelda S.M., Granot Z., Fridlender Z.G. (2014). Neutrophils recruit regulatory T-cells into tumors via secretion of CCL17—A new mechanism of impaired antitumor immunity. Int. J. Cancer.

[57] Jin L., Tao H., Karachi A., Long Y., Hou A.Y., Na M., Dyson K.A., Grippin A.J., Deleyrolle L.P., Zhang W. (2019). CXCR1- or CXCR2-modified CAR T cells co-opt IL-8 for maximal antitumor efficacy in solid tumors. Nat. Commun..

[58] Sanmamed M.F., Perez-Gracia J.L., Schalper K.A., Fusco J.P., Gonzalez A., Rodriguez-Ruiz M.E., Oñate C., Perez G., Alfaro C., Martín-Algarra S. (2017). Changes in serum interleukin-8 (IL-8) levels reflect and predict response to anti-PD-1 treatment in melanoma and non-small-cell lung cancer patients. Ann. Oncol..

[59] Collins J.M., Heery C.R., Donahue R.N., Palena C., Madan R.A., Strauss J., Gatti-Mays M.E., Schlom J., Gulley J.L., Bilusic M. (2018). Phase I trial of BMS-986253, an anti-IL-8 monoclonal antibody, in patients with metastatic or unresectable solid tumors. J. Clin. Oncol..

[60] Loumaye A., de Barsy M., Nachit M., Lause P., Frateur L., van Maanen A., Trefois P., Gruson D., Thissen J.P. (2015). Role of Activin A and myostatin in human cancer cachexia. J. Clin. Endocrinol. Metab..

[61] Ding H., Zhang G., Sin K.W.T., Liu Z., Lin R.-K., Li M., Li Y.-P. (2017). Activin A induces skeletal muscle catabolism via p38β mitogen-activated protein kinase. J. Cachexia Sarcopenia Muscle.

[62] Zhou X., Wang J.L., Lu J., Song Y., Kwak K.S., Jiao Q., Rosenfeld R., Chen Q., Boone T., Simonet W.S. (2010). Reversal of cancer cachexia and muscle wasting by ActRIIB antagonism leads to prolonged survival. Cell.

[63] Loumaye A., De Barsy M., Nachit M., Lause P., Van Maanen A., Trefois P., Gruson D., Thissen J.-P. (2017). Circulating Activin A predicts survival in cancer patients. J. Cachexia Sarcopenia Muscle.

[64] Semitekolou M., Alissafi T., Aggelakopoulou M., Kourepini E., Kariyawasam H.H., Kay A.B., Robinson D.S., Lloyd C., Panoutsakopoulou V., Xanthou G. (2009). Activin-A induces regulatory T cells that suppress T helper cell immune responses and protect from allergic airway disease. J. Exp. Med..

[65] Ogawa K., Funaba M., Chen Y., Tsujimoto M. (2006). Activin A functions as a Th2 cytokine in the promotion of the alternative activation of macrophages. J. Immunol..

[66] Rautela J., Dagley L.F., De Oliveira C.C., Schuster I.S., Hediyeh-Zadeh S., Delconte R.B., Cursons J., Hennessy R., Hutchinson D.S., Harrison C. (2019). Therapeutic blockade of Activin-A improves NK cell function and antitumor immunity. Sci. Signal..

[67] Tao J.J., Cangemi N.A., Makker V., Cadoo K.A., Liu J.F., Rasco D.W., Navarro W.H., Haqq C.M., Hyman D.M. (2019). First-in-Human Phase I Study of the Activin A Inhibitor, STM 434, in Patients with Granulosa Cell Ovarian Cancer and Other Advanced Solid Tumors. Clin. Cancer Res..

[68] Zugmaier G., Paik S., Wilding G., Knabbe C., Bano M., Lupu R., Deschauer B., Simpson S., Dickson R.B., Lippman M. (1991). Transforming Growth Factor β1 Induces Cachexia and Systemic Fibrosis without an Antitumor Effect in Nude Mice. Cancer Res..

[69] Waning D.L., Mohammad K.S., Reiken S., Xie W., Andersson D., John S.K., Chiechi A., Wright L., Umanskaya A., Niewolna M. (2015). Excess TGF-β mediates muscle weakness associated with bone metastases in mice. Nat. Med..

[70] Lima J.D., Simoes E., de Castro G., Morais M.R., de Matos-Neto E.M., Alves M.J., Pinto N.I., Figueredo R.G., Zorn T.M., Felipe-Silva A.S. (2019). Tumour-derived transforming growth factor-β signalling contributes to fibrosis in patients with cancer cachexia. J. Cachexia Sarcopenia Muscle.

[71] Batlle E., Massagué J. (2019). Transforming Growth Factor-β Signaling in Immunity and Cancer. Immunity.

[72] Polanczyk M.J., Walker E., Haley D., Guerrouahen B.S., Akporiaye E.T. (2019). Blockade of TGF-β signaling to enhance the antitumor response is accompanied by dysregulation of the functional activity of CD4+CD25+Foxp3+ and CD4+CD25-Foxp3+ T cells. J. Transl. Med..

[73] Mariathasan S., Turley S.J., Nickles D., Castiglioni A., Yuen K., Wang Y., Kadel E.E., Koeppen H., Astarita J.L., Cubas R. (2018). TGFβ attenuates tumour response to PD-L1 blockade by contributing to exclusion of T cells. Nature.

[74] Lerner L., Tao J., Liu Q., Nicoletti R., Feng B., Krieger B., Mazsa E., Siddiquee Z., Wang R., Huang L. (2016). MAP3K11/GDF15 axis is a critical driver of cancer cachexia. J. Cachexia Sarcopenia Muscle.

[75] Johnen H., Lin S., Kuffner T., Brown D.A., Tsai V.W., Bauskin A.R., Wu L., Pankhurst G., Jiang L., Junankar S. (2007). Tumor-induced anorexia and weight loss are mediated by the TGF-beta superfamily cytokine MIC-1. Nat. Med..

[76] Zhou Z., Li W., Song Y., Wang L., Zhang K., Yang J., Zhang W., Su H., Zhang Y. (2013). Growth differentiation factor-15 suppresses maturation and function of dendritic cells and inhibits tumor-specific immune response. PLoS ONE.

[77] Roth P., Junker M., Tritschler I., Mittelbronn M., Dombrowski Y., Breit S.N., Tabatabai G., Wick W., Weller M., Wischhusen J. (2010). GDF-15 contributes to proliferation and immune escape of malignant gliomas. Clin. Cancer Res..

[78] Ratnam N.M., Peterson J.M., Talbert E.E., Ladner K.J., Rajasekera P.V., Schmidt C.R., Dillhoff M.E., Swanson B.J., Haverick E., Kladney R.D. (2017). NF-κB regulates GDF-15 to suppress macrophage surveillance during early tumor development. J. Clin. Investig..

[79] Suriben R., Chen M., Higbee J., Oeffinger J., Ventura R., Li B., Mondal K., Gao Z., Ayupova D., Taskar P. (2020). Antibody-mediated inhibition of GDF15–GFRAL activity reverses cancer cachexia in mice. Nat. Med..

[80] Gabrilovich D.I., Ostrand-Rosenberg S., Bronte V. (2012). Coordinated regulation of myeloid cells by tumours. Nat. Rev. Immunol..

[81] Ohki S., Shibata M., Gonda K., Machida T., Shimura T., Nakamura I., Ohtake T., Koyama Y., Suzuki S., Ohto H. (2012). Circulating myeloid-derived suppressor cells are increased and correlate to immune suppression, inflammation and hypoproteinemia in patients with cancer. Oncol. Rep..

[82] Cuenca A.G., Cuenca A.L., Winfield R.D., Joiner D.N., Gentile L., Delano M.J., Kelly-Scumpia K.M., Scumpia P.O., Matheny M.K., Scarpace P.J. (2014). Novel role for tumor-induced expansion of myeloid-derived cells in cancer cachexia. J. Immunol..

[83] Strauss L., Mahmoud M.A.A., Weaver J.D., Tijaro-Ovalle N.M., Christofides A., Wang Q., Pal R., Yuan M., Asara J., Patsoukis N. (2020). Targeted deletion of PD-1 in myeloid cells induces antitumor immunity. Sci. Immunol..

[84] Paunescu V., Bojin F.M., Tatu C.A., Gavriliuc O.I., Rosca A., Gruia A.T., Tanasie G., Bunu C., Crisnic D., Gherghiceanu M. (2011). Tumour-associated fibroblasts and mesenchymal stem cells: More similarities than differences. J. Cell. Mol. Med..

[85] Roberts E.W., Deonarine A., Jones J.O., Denton A.E., Feig C., Lyons S.K., Espeli M., Kraman M., McKenna B., Wells R.J. (2013). Depletion of stromal cells expressing fibroblast activation protein-α from skeletal muscle and bone marrow results in cachexia and anemia. J. Exp. Med..

[86] Kir S., Spiegelman B.M. (2016). Cachexia & Brown Fat: A Burning Issue in Cancer. Trends Cancer.

[87] Ziani L., Chouaib S., Thiery J. (2018). Alteration of the Antitumor Immune Response by Cancer-Associated Fibroblasts. Front. Immunol..

[88] Tan B.H.L., Fladvad T., Braun T., Vigano A., Strasser F., Deans D.A.C., Skipworth R.J.E., Solheim T.S., Damaraju S., Ross J.A. (2012). P-selectin genotype is associated with the development of cancer cachexia. EMBO Mol. Med..

[89] Johns N., Stretch C., Tan B.H., Solheim T.S., Sørhaug S., Stephens N.A., Gioulbasanis I., Skipworth R.J., Deans D.C., Vigano A. (2017). New genetic signatures associated with cancer cachexia as defined by low skeletal muscle index and weight loss. J. Cachexia Sarcopenia Muscle.

[90] Ley K., Kansas G.S. (2004). Selectins in T-cell recruitment to non-lymphoid tissues and sites of inflammation. Nat. Rev. Immunol..

[91] Borsig L., Wong R., Hynes R.O., Varki N.M., Varki A. (2002). Synergistic effects of L- and P-selectin in facilitating tumor metastasis can involve non-mucin ligands and implicate leukocytes as enhancers of metastasis. Proc. Natl. Acad. Sci. USA.

[92] Tinoco R., Carrette F., Barraza M.L., Otero D.C., Magaña J., Bosenberg M.W., Swain S.L., Bradley L.M. (2016). PSGL-1 Is an Immune Checkpoint Regulator that Promotes T Cell Exhaustion. Immunity.

[93] Takeshige K., Baba M., Tsuboi S., Noda T., Ohsumi Y. (1992). Autophagy in yeast demonstrated with proteinase-deficient mutants and conditions for its induction. J. Cell. Biol..

[94] Aversa Z., Pin F., Lucia S., Penna F., Verzaro R., Fazi M., Colasante G., Tirone A., Fanelli F.R., Ramaccini C. (2016). Autophagy is induced in the skeletal muscle of cachectic cancer patients. Sci. Rep..

[95] Pigna E., Berardi E., Aulino P., Rizzuto E., Zampieri S., Carraro U., Kern H., Merigliano S., Gruppo M., Mericskay M. (2016). Aerobic Exercise and Pharmacological Treatments Counteract Cachexia by Modulating Autophagy in Colon Cancer. Sci. Rep..

[96] Pettersen K., Andersen S., Degen S., Tadini V., Grosjean J., Hatakeyama S., Tesfahun A.N., Moestue S., Kim J., Nonstad U. (2017). Cancer cachexia associates with a systemic autophagy-inducing activity mimicked by cancer cell-derived IL-6 trans-signaling. Sci. Rep..

[97] Rosenfeldt M.T., Ryan K.M. (2011). The multiple roles of autophagy in cancer. Carcinogenesis.

[98] Randow F., Münz C. (2012). Autophagy in the regulation of pathogen replication and adaptive immunity. Trends Immunol..

[99] Hahn T., Akporiaye E.T. (2013). α-TEA as a stimulator of tumor autophagy and enhancer of antigen cross-presentation. Autophagy.

[100] Pua H.H., Guo J., Komatsu M., He Y.-W. (2009). Autophagy is essential for mitochondrial clearance in mature T lymphocytes. J. Immunol..

[101] Wei J., Long L., Yang K., Guy C., Shrestha S., Chen Z., Wu C., Vogel P., Neale G., Green D.R. (2016). Autophagy enforces functional integrity of regulatory T cells by coupling environmental cues and metabolic homeostasis. Nat. Immunol..

[102] Liu K., Zhao E., Ilyas G., Lalazar G., Lin Y., Haseeb M., E Tanaka K., Czaja M.J. (2015). Impaired macrophage autophagy increases the immune response in obese mice by promoting proinflammatory macrophage polarization. Autophagy.

[103] Parker K.H., Horn L.A., Ostrand-Rosenberg S. (2016). High-mobility group box protein 1 promotes the survival of myeloid-derived suppressor cells by inducing autophagy. J. Leukoc. Biol..

[104] Kichenadasse G., Miners J.O., Mangoni A.A., Rowland A., Hopkins A.M., Sorich M.J. (2019). Association Between Body Mass Index and Overall Survival with Immune Checkpoint Inhibitor Therapy for Advanced Non-Small Cell Lung Cancer. JAMA Oncol..

[105] Martini D.J., Kline M.R., Liu Y., Shabto J.M., Williams M.A., Khan A.I., Lewis C., Collins H., Akce M., Kissick H.T. (2020). Adiposity may predict survival in patients with advanced stage cancer treated with immunotherapy in phase 1 clinical trials. Cancer.

[106] Shiroyama T., Nagatomo I., Koyama S., Hirata H., Nishida S., Miyake K., Fukushima K., Shirai Y., Mitsui Y., Takata S. (2019). Impact of sarcopenia in patients with advanced non–small cell lung cancer treated with PD-1 inhibitors: A preliminary retrospective study. Sci. Rep..

[107] Roch B., Coffy A., Jean-Baptiste S., Palaysi E., Daures J.-P., Pujol J.-L., Bommart S. (2020). Cachexia—Sarcopenia as a determinant of disease control rate and survival in non-small lung cancer patients receiving immune-checkpoint inhibitors. Lung Cancer.

[108] Chu M.P., Li Y., Ghosh S., Sass S., Smylie M., Walker J., Sawyer M.B. (2020). Body composition is prognostic and predictive of ipilimumab activity in metastatic melanoma. J. Cachexia Sarcopenia Muscle.

